# Commentary: Atrial Rotor Dynamics Under Complex Fractional Order Diffusion

**DOI:** 10.3389/fphys.2018.01386

**Published:** 2018-10-04

**Authors:** Alfonso Bueno-Orovio

**Affiliations:** Department of Computer Science, University of Oxford, Oxford, United Kingdom

**Keywords:** cardiac tissue, structural heterogeneity, electrical propagation, fractional diffusion, wavefront curvature

Even at healthy states, cardiac tissue conforms one of the most representative cases of a highly heterogeneous and composite biological medium, whose spatial complexity has for long been known to modulate electrical conduction (Frank and Langer, 1974; Spach et al., 1981). How to capture its intricate structural heterogeneity at a tractable cost remains an open challenge in computational physiology and medicine, as traditional approaches such as the monodomain or bidomain equations inherently assume that the tissue behaves as an averaged syncytium with negligible contribution of its composite microstructure.

To overcome some of these limitations, we recently pioneered the use of fractional diffusion for the description of cardiac conduction (Bueno-Orovio et al., 2014b). Our proposed framework took the form

(1) $$\begin{array}{l} \partial_{t}V=-{\left(-\nabla \cdot \text{D}\nabla V\right)}^{\alpha /2}-\frac{1}{C_{m}}\left(I_{\text{ion}}-I_{\text{stim}}\right), \end{array}$$

where −(−∇ · **D**∇*V*)^α/2^ is the so-called fractional Laplacian of real order 1 < α ≤ 2. For α = 2, the model clearly recovers the standard monodomain equation, and could equally be extended to the bidomain setting. The well-founded potential theory around the fractional Laplacian allowed us to establish its biophysical interpretation, showing it represents the modulation of the electrical field of a homogeneous conductor by the secondary electrical sources associated with its inhomogeneities. The model further helped elucidating formerly unrelated effects of tissue microstructure on cardiac conduction, including widespread of the action potential foot during depolarization, action potential shortening along the activation pathway, and the modulated dispersion of repolarization. Experimentally, the model has been supported by diffusion spectrum imaging in *ex-vivo* hearts, indicating fractional diffusion metrics as indices of myocardial microstructure (Bueno-Orovio et al., 2016), as well as by high-resolution optical mapping on cardiac tissue preparations, demonstrating fractional scaling in the propagation of the cardiac wavelength (Loppini et al., 2018).

In the work under comment, Ugarte et al. build and expand on these ideas to present a two-dimensional isotropic fractional diffusion framework of complex order, of the form

(2) $$\begin{array}{l} \partial_{t}V=\kappa_{\gamma }\left(H_{x}^{\gamma }V+H_{y}^{\gamma }V\right)-\frac{1}{C_{m}}\left(I_{\text{ion}}-I_{\text{stim}}\right), \end{array}$$

with operators $H_{x}^{\gamma }$ and $H_{y}^{\gamma }$ involving pairs of complex-conjugate fractional derivatives defined by

(3) $$\begin{array}{l} H_{x}^{\gamma }V=-\frac{1}{2}\left[{\left(-\partial_{x}^{2}V\right)}^{\gamma /2}+{\left(-\partial_{x}^{2}V\right)}^{\overset{-}{\gamma }/2}\right], \end{array}$$

where γ = α + *jβ* is the complex fractional order, and $\overset{-}{\gamma }$ its complex conjugate. The authors' interpretation of their complex-order model newly builds on potential theory, which connects potential distributions over fractal domains and the complex-order fractional Laplacian. Indeed, the inclusion of the imaginary part β implies that cardiac tissue must satisfy a discrete-scale fractal structure (self-similarity at discrete scales). Whilst such a self-similarity could perhaps be arguable for the main volumetric constituents of cardiac tissue (cardiomyocytes), other components might very well exhibit a fractal structure (e.g., microvasculature). Importantly, such a complex-order fractional framework holds a great potential for consideration of structural remodeling, shall the associated structures (e.g. fibrotic clefts) are proved to have a fractal nature.

However, an important limitation of Ugarte et al. (2018) is that their proposed model is not consistent with the fractional Laplacian in which the authors base their analysis. Taking β = 0 for simplicity, Equations (2), (3) then reduce to

(4) $$\begin{array}{l} \partial_{t}V=-\kappa_{\alpha }\left({\left(-\partial_{x}^{2}V\right)}^{\alpha /2}+{\left(-\partial_{y}^{2}V\right)}^{\alpha /2}\right)-\frac{1}{C_{m}}\left(I_{\text{ion}}-I_{\text{stim}}\right), \end{array}$$

known as a fractional Riesz operator (fractional derivatives independently applied in each spatial coordinate). Conversely, under two-dimensional isotropic conditions, the fractional Laplacian model given by Equation (1) becomes

(5) $$\begin{array}{l} \partial_{t}V=-\kappa_{\alpha }{\left(-\partial_{x}^{2}V-\partial_{y}^{2}V\right)}^{\alpha /2}-\frac{1}{C_{m}}\left(I_{\text{ion}}-I_{\text{stim}}\right), \end{array}$$

where for clarity the same notation κ_α_ has been used for the equivalent diffusion coefficient. Comparing (4) and (5), it becomes evident that the proposed fractional model is only equivalent to the fractional Laplacian under the standard diffusion case, given by α = 2.

The implications of these subtle but important discrepancies on cardiac conduction are exemplified in Figure 1. Simulations illustrate isotropic conduction for both models under decreasing fractional order α, with ion dynamics described for simplicity by Fenton and Karma (1998). Whereas the fractional Laplacian (Figure 1A) correctly replicates for all α the circular propagation patterns observed on isotropic cardiac monolayers as the simplest yet inhomogeneous *in-vitro* model of cardiac tissue (Badie and Bursac, 2009; Bian et al., 2014; Molitoris et al., 2016), the fractional Riesz operator (Figure 1B) induces increasingly larger curvature artifacts on wavefront conduction for decreasing α. Such curvature artifacts indeed translate into the results of Ugarte et al. (2018), as evidenced by their square-like spiral wavefronts and rotor trajectories. Given the well-known curvature-related modulation of conduction velocity and therefore wavefront-waveback interactions (Fast and Kléber, 1997; Comtois and Vinet, 1999; Comtois et al., 2005; Kadota et al., 2012), their results on vulnerability to re-entry and associated rotor biomarkers thus must be cautiously interpreted.

**Figure 1:**
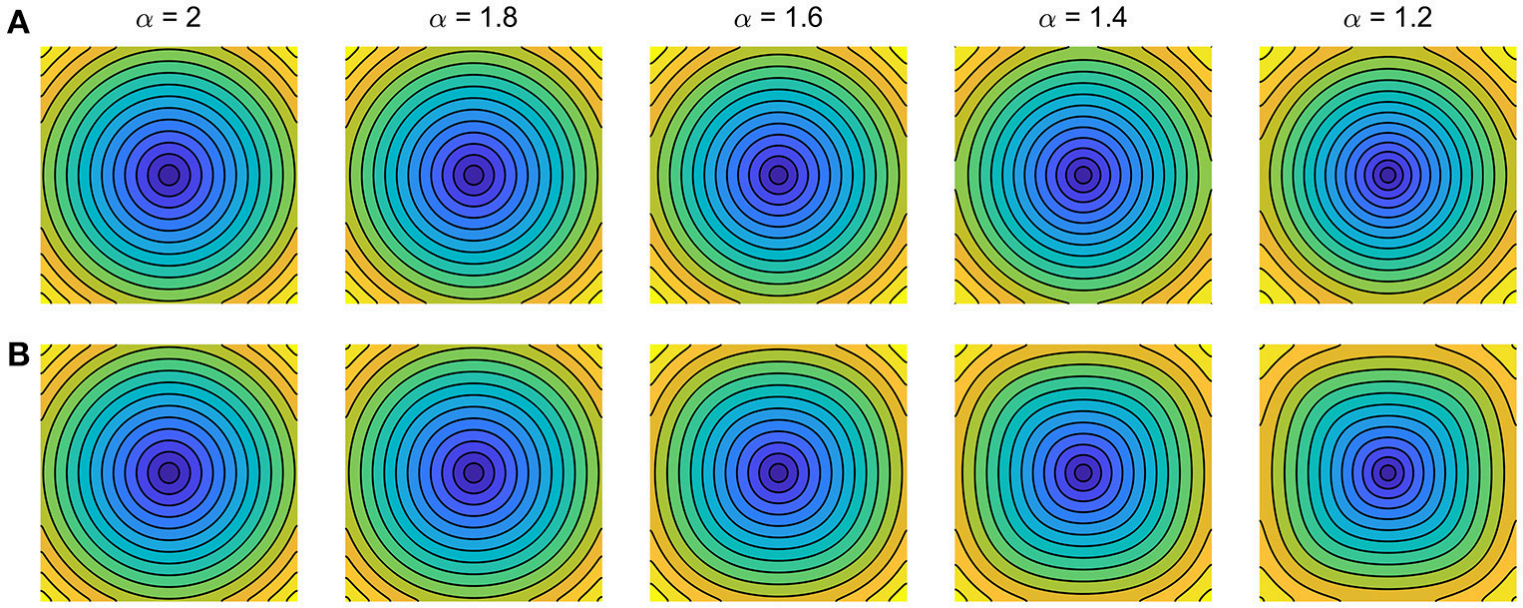
Impact of fractional diffusion operators on cardiac conduction for decreasing order α. Activation maps for central domain stimulation are shown (5 ms separation isochrones). **(A)** Fractional Laplacian (Bueno-Orovio et al., 2014b). **(B)** Fractional Riesz operator (Ugarte et al., 2018). Diffusion coefficients were optimized to match standard diffusion conduction velocity at the center of 5 cm fiber strands. Ionic term: Fenton–Karma (modified Beeler–Reuter) dynamics; domain size: 5 × 5 cm; space discretisation: 512 × 512 points; time resolution: 0.025 ms.

It is nevertheless relevant to note that more squared propagation patterns have been reported in both optical mapping (Koura et al., 2002; de Diego et al., 2011) and computational (He and Liu, 2010) studies. This was however under marked anisotropic conduction, not accounted in the isotropic model by Ugarte et al. (2018). In addition, fractional Riesz operators have been also used in modeling electrical propagation (Liu et al., 2013, 2015; Zeng et al., 2014). Such works, more centered in numerical analysis than in gaining physiological insights, might be additionally contributing to spreading the inconsistencies between these two types of fractional diffusion operators. Finally, a too coarse spatial resolution for atrial dynamics compared to previous studies (Wilhelms et al., 2013) could also contribute to partially unresolved re-entrant patterns. Although minimized by the high-order approach on which the authors base their numerical methods (Bueno-Orovio et al., 2014a), allowing considerably larger space steps than traditional stencils, this aspect certainly deserves further consideration.

As previously discussed, the ideas presented in Ugarte et al. (2018) hold a great potential for advancing the field of fractional diffusion applied to cardiac tissue, in order to promote our understanding of the role of tissue microstructure and structural remodeling in modulating wavefront propagation. However, this contribution raises awareness on the definition of suitable fractional diffusion models, exemplifying that simply recovering standard diffusion for a specific value of the considered tissue parameters is not a sufficient condition for realistic cardiac conduction. In this regard, frameworks that are consistent with the fractional Laplacian (Bueno-Orovio et al., 2014b; Cusimano et al., 2015; Cusimano and Gerardo-Giorda, 2018) seem a more suitable modeling approach to correctly capture the characteristic electrotonic loading of cardiac tissue.

## Author contributions

The author confirms being the sole contributor of this work and has approved it for publication.

### Conflict of interest statement

The author declares that the research was conducted in the absence of any commercial or financial relationships that could be construed as a potential conflict of interest.

## References

[B1] Badie N. Bursac N. (2009). Novel micropatterned cardiac cell cultures with realistic ventricular microstructure. Biophys. J. 96, 3873–3885. 10.1016/j.bpj.2009.02.019 19413993 PMC3297758

[B2] Bian W. Jackman C. P. Bursac N. (2014). Controlling the structural and functional anisotropy of engineered cardiac tissues. Biofabrication 6:024109. 10.1088/1758-5082/6/2/024109 24717534 PMC4040155

[B3] Bueno-Orovio A. Kay D. Burrage K. (2014a). Fourier spectral methods for fractional-in-space reaction-diffusion equations. BIT Numer. Math. 54, 937–954. 10.1186/s40064-016-3295-x 27722061 PMC5033804

[B4] Bueno-Orovio A. Kay D. Grau V. Rodriguez B. Burrage K. (2014b). Fractional diffusion models of cardiac electrical propagation: role of structural heterogeneity in dispersion of repolarization. J. R. Soc. Interface 11:20140352. 10.1098/rsif.2014.0352 24920109 PMC4208367

[B5] Bueno-Orovio A. Teh I. Schneider J. E. Burrage K. Grau V. (2016). Anomalous diffusion in cardiac tissue as an index of myocardial microstructure. IEEE Trans. Med. Imaging 35, 2200–2207. 10.1109/TMI.2016.2548503 27164578

[B6] Comtois P. Kneller J. Nattel S. (2005). Of circles and spirals: bridging the gap between the leading circle and spiral wave concepts of cardiac reentry. Europace 7, S10–S20. 10.1016/j.eupc.2005.05.011 16102499

[B7] Comtois P. Vinet A. (1999). Curvature effects on activation speed and repolarization in an ionic model of cardiac myocytes. Phys. Rev. E 60:4619. 11970323 10.1103/physreve.60.4619

[B8] Cusimano N. Bueno-Orovio A. Turner I. Burrage K. (2015). On the order of the fractional Laplacian in determining the spatio-temporal evolution of a space-fractional model of cardiac electrophysiology. PLoS ONE 10:e0143938. 10.1371/journal.pone.0143938 26629898 PMC4668072

[B9] Cusimano N. Gerardo-Giorda L. (2018). A space-fractional monodomain model for cardiac electrophysiology combining anisotropy and heterogeneity on realistic geometries. J. Comput. Phys. 362, 409–424. 10.1016/j.jcp.2018.02.034

[B10] de Diego C. Chen F. Xie Y. Pai R. K. Slavin L. Parker J.. (2011). Anisotropic conduction block and reentry in neonatal rat ventricular myocyte monolayers. Am. J. Physiol. Heart Circ. Physiol. 300, H271–H278. 10.1152/ajpheart.00758.2009 21037233 PMC3023258

[B11] Fast V. G. Kléber A. G. (1997). Role of wavefront curvature in propagation of cardiac impulse. Cardiovasc. Res. 33, 258–271. 10.1016/S0008-6363(96)00216-7 9074688

[B12] Fenton F. Karma A. (1998). Vortex dynamics in three-dimensional continuous myocardium with fiber rotation: filament instability and fibrillation. Chaos 8, 20–47. 10.1063/1.166311 12779708

[B13] Frank J. S. Langer G. A. (1974). The myocardial interstitium: its structure and its role in ionic exchange. J. Cell Biol. 60, 586–601. 10.1083/jcb.60.3.586 4824287 PMC2109249

[B14] He Z. Z. Liu J. (2010). Effect of cardiac tissue anisotropy on three-dimensional electrical action potential propagation. Mod. Phys. Lett. B. 24, 1847–1853. 10.1142/S0217984910024237

[B15] Kadota S. Kay M. W. Magome N. Agladzea K. (2012). Curvature-dependent excitation propagation in cultured cardiac tissue. JETP Lett. 94, 824–830. 10.1134/S0021364011230044 26705369 PMC4687754

[B16] Koura T. Hara M. Takeuchi S. Ota K. Okada Y. Miyoshi S.. (2002). Anisotropic conduction properties in canine atria analyzed by high-resolution optical mapping: preferential direction of conduction block changes from longitudinal to transverse with increasing age. Circulation 105, 2092–2098. 10.1161/01.CIR.0000015506.36371.0D 11980690

[B17] Liu F. Turner I. Anh V. Yang Q. Burrage K. (2013). A numerical method for the fractional Fitzhugh–Nagumo monodomain model. ANZIAM J. 54, C608–C629. 10.21914/anziamj.v54i0.6372

[B18] Liu F. Zhuang P. Turner I. Anh V. Burrage K. (2015). A semi-alternating direction method for a 2-D fractional FitzHugh–Nagumo monodomain model on an approximate irregular domain. J. Comput. Phys. 293, 252–263. 10.1016/j.jcp.2014.06.001

[B19] Loppini A. Gizzi A. Cherubini C. Cherry E. M. Fenton F. H. Filippi S. (2018). Spatiotemporal correlation uncovers fractional scaling in cardiac tissue. arXiv [preprint] arXiv:1806.04507.

[B20] Molitoris J. M. Paliwal S. Sekar R. B. Blake R. Park J. Trayanova N. A.. (2016). Precisely parameterized experimental and computational models of tissue organization. Integr. Biol. 8, 230–242. 10.1039/C5IB00270B 26822672 PMC4831076

[B21] Spach M. S. Miller W. T. III Geselowitz D. B. Barr R. C. Kootsey J. M. Johnson E. A. (1981). The discontinuous nature of propagation in normal canine cardiac muscle. Evidence for recurrent discontinuities of intracellular resistance that affect the membrane currents. Circ. Res. 48, 39–54. 10.1161/01.RES.48.1.39 7438345

[B22] Ugarte J. P. Tobón C. Lopes A. M. Tenreiro Machado J. A. (2018). Atrial rotor dynamics under complex fractional order diffusion. Front. Physiol. 9:975. 10.3389/fphys.2018.00975 30087620 PMC6066719

[B23] Wilhelms M. Hettmann H. Maleckar M. M. Koivumäki J. T. Dössel O. Seemann G. (2013). Benchmarking electrophysiological models of human atrial myocytes. Front. Physiol. 3:487. 10.3389/fphys.2012.00487 23316167 PMC3539682

[B24] Zeng F. Liu F. Li C. Burrage K. Turner I. Anh V. (2014). A Crank–Nicolson ADI spectral method for a two-dimensional Riesz space fractional nonlinear reaction-diffusion equation. SIAM J. Numer. Anal. 52, 2599–2622. 10.1137/130934192

